# CircRNAs: versatile players and new targets in organ fibrosis

**DOI:** 10.1186/s12964-023-01051-1

**Published:** 2023-05-02

**Authors:** Lei Wei, Limin Liu, Ming Bai, Xiaoxuan Ning, Shiren Sun

**Affiliations:** 1 Department of Nephrology, Xijing Hospital, The Fourth Military Medical University, No. 127 Changle West Road, Xi’an, Shaanxi China; 2 School of Medicine, Northwest University, 229 Taibai North Road, Xi’an, 710032 Shaanxi China; 3 Department of Geriatrics, Xijing Hospital, Fourth Military Medical University, No. 127 Changle West Road, Xi’an, 710032 Shaanxi China

**Keywords:** circRNA, Fibrosis, miRNA sponge, Fibroblasts, Exosomal circRNA biomarkers, Therapeutics

## Abstract

Organ fibrosis can occur in virtually all major organs with relentlessly progressive and irreversible progress, ultimately resulting in organ dysfunction and potentially death. Unfortunately, current clinical treatments cannot halt or reverse the progression of fibrosis to end-stage organ failure, and thus, advanced antifibrotic therapeutics are urgently needed. In recent years, a growing body of research has revealed that circular RNAs (circRNAs) play pivotal roles in the development and progression of organ fibrosis through highly diverse mechanisms of action. Thus, manipulating circRNAs has emerged as a promising strategy to mitigate fibrosis across different organ types. In this review, we systemically summarize the current state of knowledge about circRNA biological properties and the regulatory mechanisms of circRNAs. A comprehensive overview of major fibrotic signaling pathways and representative circRNAs that are known to modulate fibrotic signals are outlined. Then, we focus on the research progress of the versatile functional roles and underlying molecular mechanisms of circRNAs in various fibrotic diseases in different organs, including the heart, liver, lung, kidney and skin. Finally, we offer a glimpse into the prospects of circRNA-based interference and therapy, as well as their utilization as biomarkers in the diagnosis and prognosis of fibrotic diseases.

**Video abstract**

**Supplementary Information:**

The online version contains supplementary material available at 10.1186/s12964-023-01051-1.

## Key points

- Aberrant expression of circRNA has been described in fibrotic states affecting different organs, including the lung, heart, liver, kidney and skin. Some circRNAs are closely associated with fibrogenesis through their effects on various fibrotic pathways.
- CircRNAs have anti- or pro-fibrotic effects depending on the downstream targeted molecules and pathways and might contribute to the regulation of inflammation, apoptosis, fibroblast activation and proliferation, and extracellular matrix accumulation.
- A substantial body of research has mainly focused on the well-known microRNA sponge role of circRNAs. Recently, new mechanisms, including interactions with proteins, coding capacities, and extracellular exosomal circRNAs, have been uncovered.
- Much research has examined fibrotic effector cells, particularly fibroblasts and myofibroblasts. The circ-transcriptome in other cell types, including epithelial, endothelial, and immune cells involved in fibrosis, needs to be profiled.
- Further work is needed to identify and validate circRNAs as biomarkers of the development and progression of fibrotic diseases. Although circRNAs are promising antifibrotic therapeutic targets, their clinical use remains far off. Purification of synthetic circRNAs and their safety are key issues that need to be solved before they can be successfully translated into clinical settings.

**Supplementary Information:**

The online version contains supplementary material available at 10.1186/s12964-023-01051-1.

## Introduction

Organ fibrosis is the final common pathological pathway of many chronic diseases and is characterized by various injuries and insults with maladaptive tissue repair, followed by excessive collagen deposition into the extracellular matrix (ECM). Structural alterations lead to tissue stiffness, vascular rarefaction, hypoxia, proliferation, differentiation and activation of fibroblasts and an inflammatory cascade that reinforces a vicious cycle of tissue destruction. Fibrosis can affect virtually every organ system, including the liver, heart, kidney, pulmonary system, and skin. The combined annual incidence of major fibrosis-related diseases is approximately 4968 per 100,000 person-years and has been estimated to contribute to 45% of annual deaths worldwide [1]. Despite imposing an enormous socioeconomic burden globally, there is a lack of available treatments to mitigate, halt or reverse fibrotic progression. Hence, it is imperative to explore the mechanisms underlying organ fibrosis and find potential new targets for antifibrotic therapies. Circular RNAs (circRNAs) are a large class of noncoding RNA molecules identified over the last decade that have recently been found to exert their pathophysiological roles through multidimensional mechanisms, including at the transcriptional, epigenetic, translational, and posttranslational levels [2]. In particular, circRNAs play critical roles in the development and progression of fibrosis [3], and manipulating circRNAs has shown promising prospects as ideal biomarkers of fibrosis and antagonists of fibrotic disorders in vivo. In this review, we provide an update on the versatile mechanisms linking circRNAs to fibrosis in different organs, and we uncover the potential of circRNAs as therapeutic targets and biomarkers.

### Properties of circRNAs

**Fig. 1:**
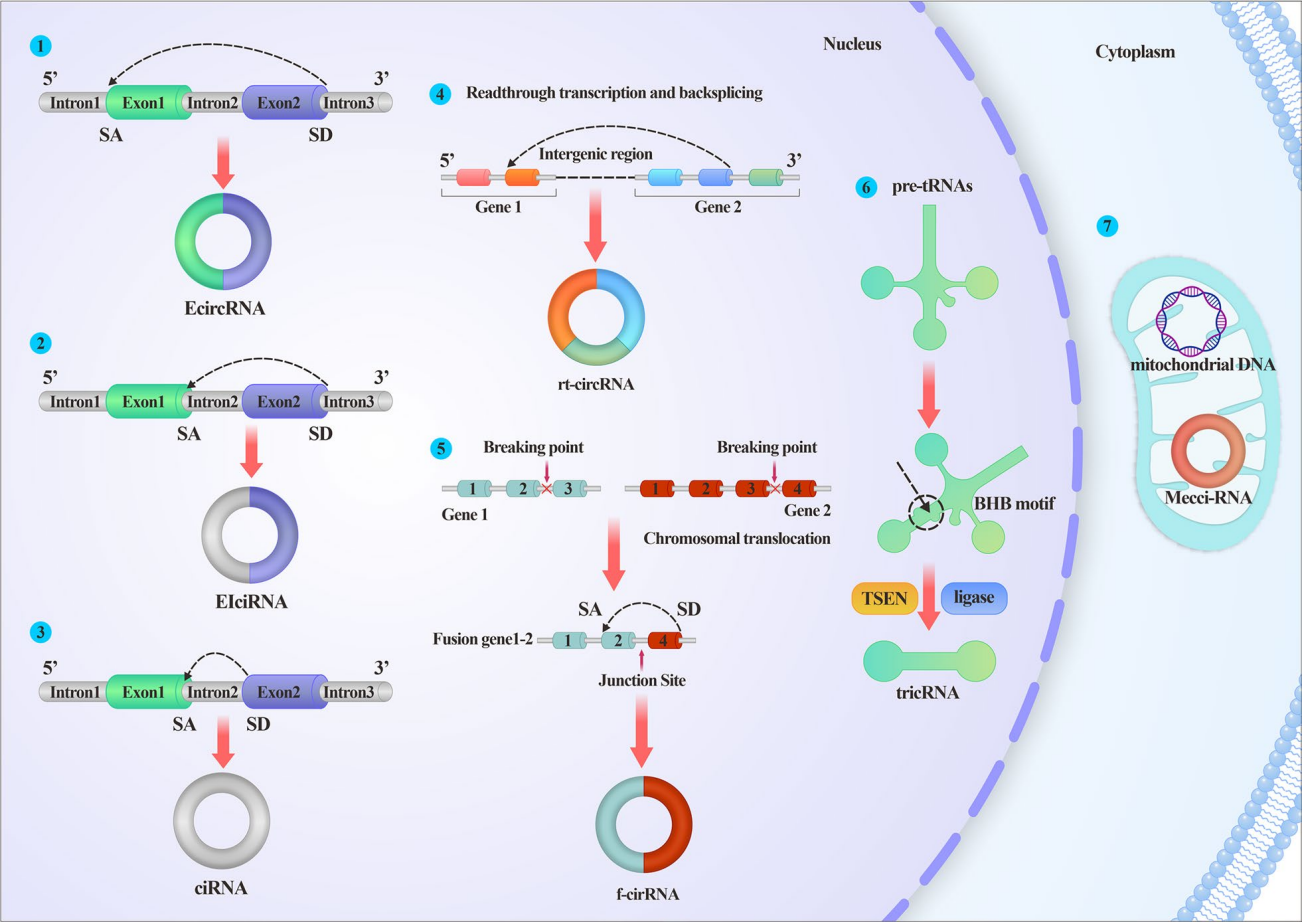
Illustrations of different categories of circRNA. ①EcircRNA is formed by the back-splicing of exons; ②EIciRNA is circularized with introns “retained” between exons; ③ciRNA is formed by the back-splicing of the intron; ④rt-circRNA is a hybrid circle that includes coding exons from two adjacent and similarly oriented genes. ⑤f-circRNA is generated from transcribed exons of distinct genes affected by translocations. ⑥TricRNA is derived from pretRNA, which is recognized and cleaved by TSEN and produces 5’-OH and a 2’,3’-cyclic phosphate at each site of cleavage. Ligation of these two cleavage sites generates a circle. ⑦mecci-RNA refers to mtDNA-encoded or mitochondria-located circRNAs. *SA* splicing acceptor, *SD* splicing donor, *EcirRNA* exon circRNA, *EIciRNA* exon‒intron circRNA, *ciRNA* intronic circRNA, *rt-circRNA* read-through circRNA, *f-circRNA* fused circRNA, *tricRNA* tRNA intronic circular RNA, *mecciRNA* mitochondrial circRNA

Sanger et al. first isolated and termed circRNAs in plant viruses and animal hepatitis almost half a century ago. However, these newly discovered circRNAs were simply associated with ‘scrambled exons’ or ‘mis-splicing’ until 2013, when two articles published in Nature [4, 5] identified the sponge-like function of microRNAs (miRNAs). Since then, circRNAs have become a research hotspot. CircRNAs are characterized as peculiar, single-stranded covalently closed RNA loops that spatially form 3D structures and interact with components, including RNA-binding proteins (RBPs), DNA or RNA. They are without 5’-caps and 3’-poly(A) tails and are thus resistant to exonuclease degradation and quite stable relative to their linear cognates. CircRNAs exert functions independently of their parental transcripts, and they are spatial-temporal, tissue- or cell-, and developmental stage-specific. Based on their origins and circularization patterns, circRNAs are categorized into at least seven types: intronic circRNAs (ciRNAs), exon‒intron circRNAs (EIciRNAs), exon circRNAs, tRNA intronic circular RNAs, mitochondrial circRNAs (mecciRNAs), read-through circRNAs (from exons between neighboring genes on the same strand), and fused circRNAs (fused exons between two remote genes) (Figs. 1) [2]. EIciRNAs and ciRNAs are sequestered in the nucleus, exon circRNAs are mostly exported to the cytoplasm, fused circRNAs are localized in the nucleus and cytoplasm, and mecciRNAs are located in the mitochondria and cytoplasm [6]. Localization is closely linked to function. ciRNAs and EIciRNAs regulate transcription via interaction with the U1 small nuclear ribonucleoprotein (snRNP). Fused circRNAs formed by cancer-associated chromosomal translocations are usually oncogenes involved in tumorigenesis. mecciRNAs regulate reactive oxygen species (ROS) in mitochondria. The function of read-through circRNAs is not yet well understood. Exon circRNAs act as sponges for proteins or miRNAs, scaffolds or templates for translation, thus playing more diverse roles than other types.

### Biogenesis, transportation and degradation

**Fig. 2:**
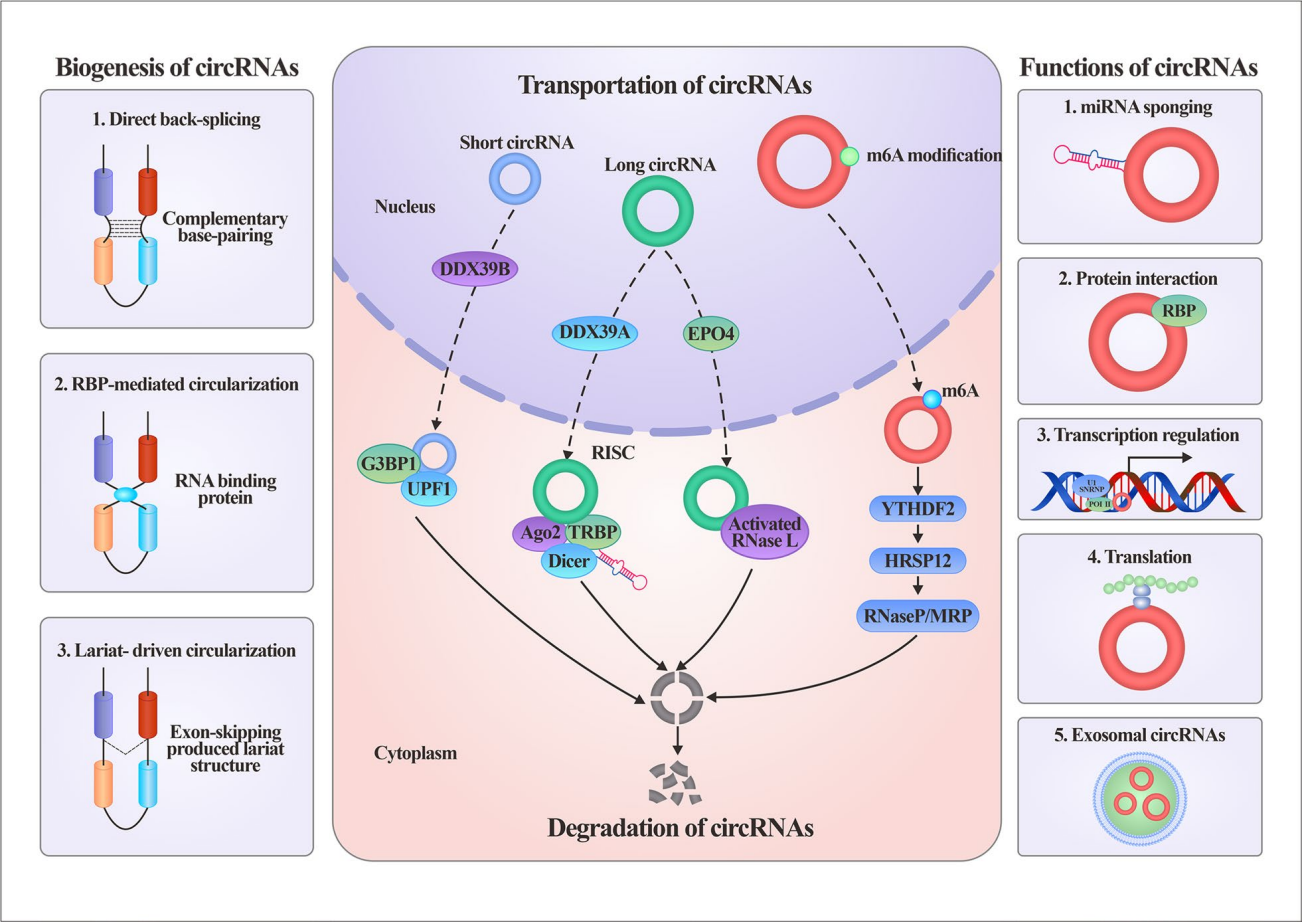
The biogenesis, transportation, degradation and functions of circRNA. The left panel describes the three mechanisms of biogenesis of circRNAs, including complementary base-pairing driven, RBP-driven and lariat-driven circularization. The middle panel illustrates the transportation and degradation of circRNAs. The right panel briefly summarizes the modes of action of circRNAs. *DDX39A* DEAD-box protein 39 A, *EPO4* Exportin 4, *G3BP1* Ras-GTPase-activating SH3 domain-binding-proteins 1, *UPF1* Upframeshift 1, *RISC* RNA-induced silencing complex, *Ago* Argonaute, *TRBP* Transactivation response (TAR) RNA-binding protein, *YTHDF* YTH domain family, *HRSP12* heat responsive protein 12, *RBP* RNA binding protein

Spliceosomes, cis-complementary sequences, epigenetic modulators and proteins are all involved in the biogenesis of circRNAs. There are three proposed mechanisms, direct back-splicing or intronic complementary sequence-driven circularization, RBP-mediated circularization, or lariat-mediated back-splicing, to decipher how circRNAs are alternatively spliced (Fig. 2, left panel) [7]. The complementary base pairing of inverted repeats flanking the exons mediates direct back-splicing. ALU repeats are the main pattern of circularization. Reverse complementary matches enriched in introns promote the formation of hairpin structures in transcripts, thus facilitating direct joining of the 5’ and 3’ splice sites. RBPs can inhibit or promote the biogenesis of circRNAs by binding specific motifs in the flanking introns, and their regulation of circRNAs is closely related to the tissue, cellular or developmental specificities of circRNAs [8]. Quaking, muscleblind, heterogeneous nuclear ribonucleoprotein L, fused-in-sarcoma, RNA-binding motif protein 20, nuclear factor 90 and nuclear factor 110 are RBPs that commonly facilitate circRNA production. DEAH-box helicase 9 and adenosine deaminase acting on RNA 1 have been reported to repress circRNA biogenesis by binding inverted repeat Alus in flanking sequences or A-to-I RNA editing activity [9]. A lariat structure is produced by exon-skipping during pre-mRNA processing, which can then undergo internal splicing to form exon circRNAs or EIcircRNAs. In addition, increased N6-methyladenosine modification (m6A) levels are associated with the production of circRNAs [10]. Writers (methyltransferases), erasers (demethylases), or readers (RBPs) modulate the circularization of m6A exons [11, 12].

The specific mechanism of circRNA transportation from the nucleus into the cytoplasm is not completely understood (Fig. 2, middle panel). The ATP-dependent RNA helicase DEAD-box protein 39 A is required for efficient nuclear export of long circRNAs, whereas DEAD-box protein 39 B controls the localization of short circRNAs [13]. Recently, Chen et al. reported that exportin 4 deficiency leads to nuclear accumulation of exon circRNAs, deleterious formation of RNA:DNA hybrids and DNA damage [14]. It has been demonstrated that m6A modification also plays pivotal roles in the nucleus–cytoplasm output of circRNAs [15].

CircRNAs are highly stable due to their resistance to exonucleases; thus, the mechanisms for their degradation remain to be fully elucidated (Fig. 2, middle panel). The binding of miRNAs to circRNAs can initiate Argonaute 2 (Ago2)-mediated circRNA degradation executed by the RNA-induced silencing complex (RISC). m6A modifications have been shown to cause widespread and diverse modulations of circRNA turnover. A subset of circRNAs containing m6A was identified as degraded by the YTHDF2-HRSP12-RNase P/MRP axes [16]. Structure-mediated RNA decay models formed by base pairing in circRNAs require the RBP UPF1 and its associated protein G3BP1, and downregulation of either UPF1 or G3BP1 causes the accumulation of circRNAs [17]. Furthermore, circRNAs can also be globally degraded by activated RNase L upon viral infection, which is required for PKR activation [18].

### Regulatory roles of circRNAs

circRNAs play essential roles in various pathophysiological conditions via interactions with numerous molecules at different levels. Their modes of action have recently been systemically reviewed elsewhere [7, 19, 20] and can be briefly summarized into five categories (Fig. 2, right panel): (A) miRNA sponges or decoys; (B) interactions with proteins; (C) regulation of transcription; (D) translation into proteins and peptides; and (E) mediation of cell‒cell communications (exosomal circRNAs). The miRNA sponge role is the most extensively investigated mechanism, but major pitfalls and controversies exist in this research field [21]. Major issues include being based solely on bioinformatics, a lack of fundamental validation, expression at relatively low levels, mismatched stoichiometry between circRNAs and miRNAs, and the harboring of a limited binding region. In contrast to other action patterns, the interactions between circRNAs and proteins are much more intricate [22]. circRNAs can change protein interaction modes, tether or sequester proteins, recruit proteins to chromatin, form circRNA–protein‒mRNA ternary complexes, and translocate or redistribute proteins. Interactions with proteins are the chief executors of life processes, and these interactions endow circRNAs with flexible and crucial functions. circRNAs regulate transcription at the initiation and elongation steps. They can serve as cis- or trans-acting elements and promote RNA Pol II transcription by interacting with U1 snRNP. In addition, circRNAs can form R loops with their cognate gene [23] or coactivate transcription factors to modulate transcription [24]. CircRNAs were previously thought to be noncoding RNAs due to their lack of a 5’ cap and 3’ poly (A) tail structure, which are necessary for cap-dependent translation. The identification of cap-independent mechanisms has revolutionized the concept that circRNAs are actually able to function as mRNAs to direct protein synthesis into peptides or proteins. Cap-independent translation can be mediated by the internal ribosome entry site-initiated pattern, m6A internal ribosome entry site-initiated patterns, and rolling translation [6]. By adopting a cell-based reporter system and using mass spectrum techniques, Fan et al. identified hundreds of circRNA-coding peptides, providing robust evidence of the coding capacity of circRNAs [25]. The new isoforms formed by noncanonical translation of circRNAs are different from their parental genes, thus opening a broad array of actions that can potentially extend well beyond mRNA-coding proteins [26].

Finally, as discussed in more detail below, circRNAs are cargoes packaged into exosomes that can mediate crosstalk involving numerous cell and organ systems [27], and exosomal circRNAs are promising biomarkers and therapeutic targets [28].

### CircRNAs are pivotal regulators involved in the molecular pathways of organ fibrosis

**Fig. 3:**
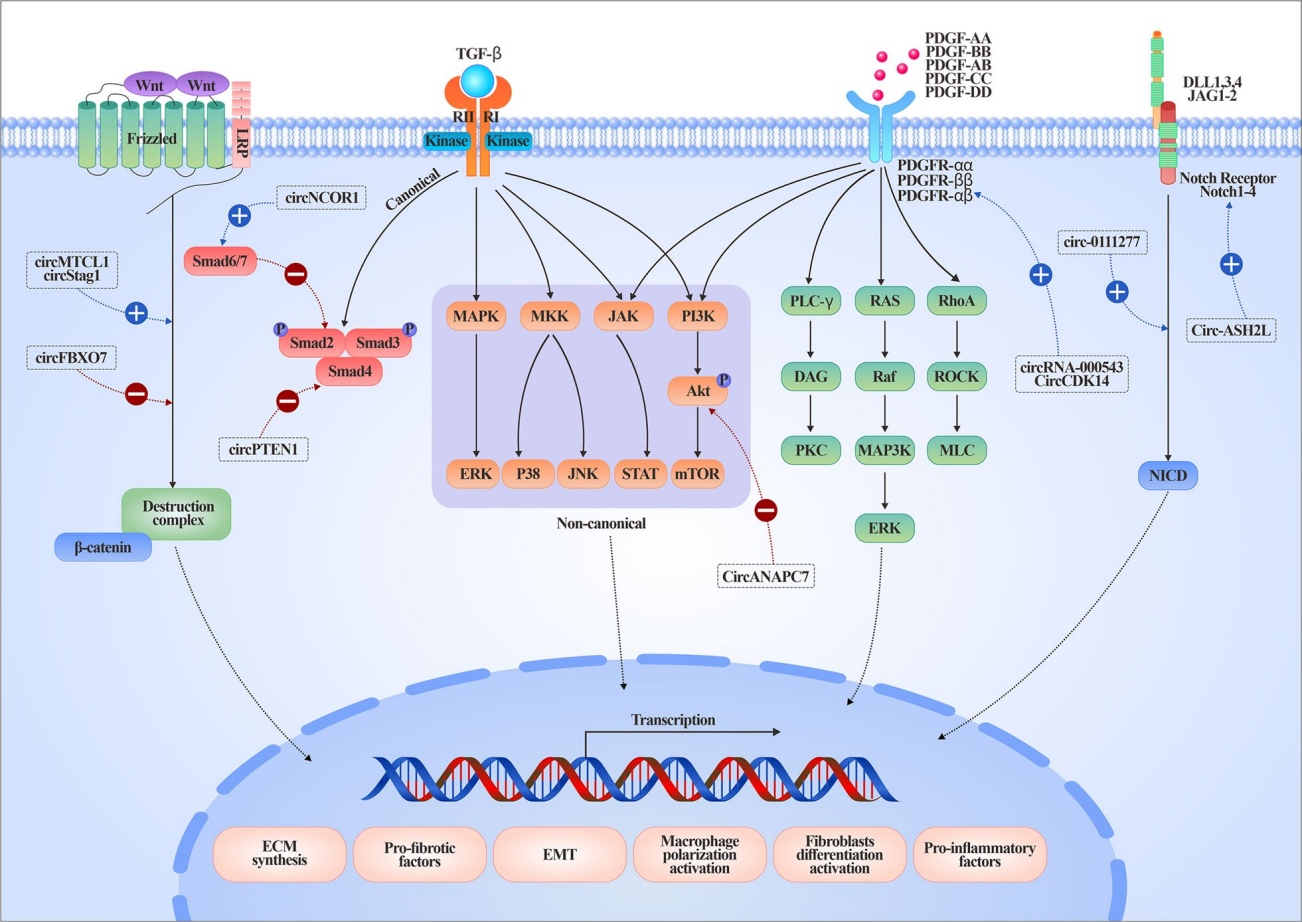
Representative circRNAs involved in core and regulatory fibrotic signaling pathways. Fibrotic signaling includes the TGF-β1, Wnt, Notch and PDGF pathways. The blue dotted line and a plus sign represent promoted effects, whereas a red dotted line and a minus sign represent inhibited effects. *EMT* Epithelial-mesenchymal transition, *ECM* Extracellular matrix

Fibrosis is a multistaged and elaborate process involving numerous cells and extra or intracellular or soluble signaling molecules (Fig. 3). The fibrotic signaling pathways are not independent of each other but communicate in an orchestrated fashion, jointly amplifying the fibrotic cascades. Recently, circRNAs have been demonstrated to be pivotal regulators involved in complicated fibrotic signaling pathways, highlighting their potential as therapeutic targets.

### Transforming growth factor β (TGF-β) signaling

TGF-β, a pluripotent growth factor, has been demonstrated to be the master regulator of fibrosis and orchestrates core and regulatory fibrotic signaling pathways. Therapeutic agents, including oligonucleotides, peptides, receptor blockers, antibodies, and small-molecule inhibitors, target the TGF-β network by inhibiting TGF-β synthesis, activation, binding to TGF-β receptors, TGF-βR function, or downstream molecules. However, these existing strategies have little impact on halting or suppressing fibrosis. Upon activation from its latent form, TGF-β binds to TGF-βRII, which subsequently dimerizes with and phosphorylates TGF-βRI. The downstream pathways of TGF-β include canonical solvated metal atom dispersed (smad)-dependent signaling and smad-independent noncanonical pathways (mitogen-activated protein kinase/extracellular signal-regulated kinase, p38 and JUN amino-terminal kinase, or phosphatidylinositol 3-kinase (PI3K/AKT)) [29]. CircANAPC7 functions as an anti-oncogene in pancreatic cancer through the miR-373/PH domain leucine-rich repeat-containing protein phosphatase 2 axis, leading to AKT dephosphorylation and TGF-β downregulation [30]. circPTEN1 binds to the MH2 domain of Smad4 to disrupt its physical interaction with Smad2/3 and blocks TGF-β signaling activation [31]. Overexpression of circNCOR1 epigenetically promotes Smad7 transcription, thereby inhibiting the TGF-β-Smad signaling pathway [32].

#### WNT signaling

WNT signaling is another core fibrotic pathway, and inhibition of Wnt signaling has antifibrotic effects on different organs. WNT pathways can be subdivided into β-catenin-dependent (also referred to as “canonical” WNT signaling) and β-catenin-independent cascades (“noncanonical” WNT signaling, including Wnt/planar cell polarity and the Wnt/calcium pathway). circFBXO7 represses Wnt/β-catenin signaling through the miR-96-5p/MTSS1 axis [33]. circMTCL1 acts as an oncogene by promoting complement C1q-binding protein-dependent ubiquitin degradation and subsequently activating Wnt/β-catenin signaling [34]. circStag1 is an osteoporosis-related circRNA that mediates the cytoplasmic translocation of human antigen R and then activates Wnt signaling pathways [35].

#### Notch signaling

Notch signaling relies on direct cell‒cell contact and is evolutionarily conserved. Membrane-bound ligands bind to Notch receptors on the target cell and activate the Notch signaling cascade. Four paralogs of Notch receptors (Notch1-4) and five different ligands (Delta-like 1 (DLL1), DLL3 and DLL4) and Jagged protein families (JAG1 and JAG2) have been described in mammals. Aberrant Notch signaling activation has been closely associated with fibrotic diseases [36]. circ_0111277 activates HTRA1/Notch-1 signaling by sponging miR-494-3p [37], whereas circ-ASH2L upregulates Notch 1 expression by serving as a sponge for miR-34a [38].

#### Platelet-derived growth factor (PDGF) signaling

PDGF has four isoforms (A, B, C and D) and forms five dimeric proteins (AA, BB, AB, CC and DD). The engagement of two PDGF receptor tyrosine kinases, PDGFRα and PDGFRβ, activates mitogen-activated protein kinase and PI3K, as well as the small RHO family GTPases involved in cell motility, SRC and other nonreceptor tyrosine kinases, and phospholipase Cγ. PDGF signaling promotes fibrosis by acting as a mesenchymal cell mitogen and by stimulating collagen contraction. circRNA_000543 [39] and CircCDK14 [40] increase PDGFRβ and PDGFRα by sponging miR-9 and miR-3938, respectively.

### The emerging roles of circRNAs in fibrotic diseases

Common diseases associated with fibrosis include chronic kidney disease (CKD), cirrhosis, hepatitis, nonalcoholic steatohepatitis, myocardial infarction, heart failure, diabetes, idiopathic pulmonary fibrosis, and scleroderma. Aberrant expression of circRNAs has been implicated in a wide variety of diseases, especially cancers, neurological disease, and metabolic disorders. Mounting evidence has outlined functional roles for circRNAs in promoting fibrotic diseases or exerting protective effects against them [41]. Here, we provide a comprehensive update of circRNA functions in different organ systems (Table 1).

**Table 1:** The summaries of representative circRNAs in different organ fibrosis

Organ	Diseases	CircRNA	Dysregulation in disease	Pathophysiological function	Mechanism of action	References
Heart	Doxorubicin- induced CF	CircNlgn	Upregulated in myocardial tissues of patients with selected congenital heart defects with cardiac overload.	Transgenic mice overexpressing circNlgn impaired cardiac function and induced CF. Silencing circNlgn decreased the effects of doxorubicin on cardiac cell activities and retarded fibrosis.	CircNlgn-derived peptide Nlgn173 could bind and activate H2AX, producing γH2AX, resulting in upregulation of IL-1b, IL-2Rb, IL-6 and EGR1/3.	[45]
Heart	CF	CircYap	Decreased in patients with cardiac hypertrophy and TAC mice.	Ectopic circYap improved heart function and alleviated fibrosis.	CircYap facilitated the binding of TPM4 with ACTG, resulting in increased inhibitory effect of TPM4 on actin polymerization.	[49]
Heart	Myocardial fibrosis	CircUbe3a	Elevated in M2M-SEV in heart of acute myocardial injury.	Impaired cardiac function and exacerbated cardiac fibrosis by promoting the proliferation, migration and differentiation.	Targeting miR-138-5p/RhoC signaling pathway.	[52]
Heart	Cardiac hepatopathy	Circslc8a1	Decreased in mice TAC heart.	Silencing circslc8a1induces cardiac hypertrophy, fibrosis, and hepatic steatosis, and over-expressing it exerts protective effects.	Interacted with mitochondrial-associated proteins, i.e. ATBP, COX5B, NUDFS2, and facilitate ATP production.	[53]
Liver	Radiation-induced liver disease	CircRSF1	Upregulated in irradiated LX2 cells	Promoted the survival and inflammatory as well as fibrotic phenotype of irradiated LX2 cells.	MiR-146a-5p/RAC1	[57]
Liver	CCl4-induced HF	CircFBXW4	Decreased in HF	Overexpression of circFBXW4 inhibited HSC proliferation and activation, induced HSC apoptosis, thereby antagonizing fibrosis.	MiR-18b-3p/FBXW7	[59]
Liver	CCl4-induced HF	CircPSD3	Down-regulated in HF	Inhibited the proliferation and activation of HSC. In vivo overexpression of circPSD3 alleviated hepatic fibrosis.	MiR-92b-3p/smad7	[60]
Liver	CCl4-induced HF	CircUbe2k	Elevated in HF.	Enhanced the activation and and prolifereation of HSC in vivo and in vitro.	MiR-149-5p/TGF-β2	[65]
Liver	NASH	CircSCAR	Decreased in HSC from NASH patients.	Inhibits mitochondrial ROS output and fibroblast activation.	Binds to ATP5B and shut down mPTP by blocking CypD-mPTP interaction.	[69]
Lung	IPF	CircANKRD42	Upregulated in peripheral blood of IPF patients.	Enhanced the mechanical stiffness. Promoted the fibroblasts differentiation, activation and migration, and ECM synthesis.	Directly targets AJUBA and YAP1, through sponging miR-324-5p and miR-136-5p, respectively, and promoted the nuclear translocation of pYAP1, therefore facilitating the transcription of genes related to mechanical stiffness.	[71]
Lung	IPF	CircTADA2A	Downregulated in IPF fibroblasts.	CircTADA2A decreased fibrogenic responses in vivo and vitro.	Targeting miR-526b/Cav1 and miR-203/Cav2 axes	[72]
Lung	Bleomycin- induced PF	CircHIPK3	Upregulated in pulmonary fibrosis.	Silencing circHIPK3 inhibited fibroblast-to-myofibroblast transition and suppressed fibroblasts proliferation.	Targeting miR-338-3p/COL1A1, SOX4, or enhancing glycolysis through miR-30a-3p/FOXK axis.	[75]
Renal	FAN	CircHIPK3	Increased in FAN	Promoted the production of profibrotic factors.	Targeting miR-30a and uprelagulating the downstream genes, including FN, TGFb1, COL1.	[81]
Renal	IRI or toxic kidney diseases	CircBNC2	Decreased in IRI or toxic kidney injuries	Ectopic circBNC2 mitigated maladaptive fibrotic repair after kidney injury by improving TEC G2/M arrest.	Translating into a 681-amino-acid protein and promoted CDK1/cyclin B1 complex formation and nuclear translocation, and alleviated TEC G2/M arrest.	[84]
Renal	IRI, UUO and patients with CKD	CircPTPN14	Increased in RF.	Silencing circPTPN14 alleviated the progression of fbrosis in kidneys subjected to IRI or UUO.	Promoting FUSP1 binding to FUSE, which enhances MYC and related fibrotic genes transcription.	[85]
Skin	Keloid tissues and keloid fibroblasts	Circ_0057452	Upregulated in keloid fibroblasts	Knockdown of circ_0057452 inhibited the proliferation, migration, invasion, and collagen synthesis and induced cell cycle arrest and apoptosis of KFs.	MiR-7-5p/GAB1	[89]
Skin	Keloid tissues and keloid fibroblasts	CircCOL5A1	Upregulated in keloid fibroblasts	CircCOL5A1 knockdown repressed the proliferation, migration, ECM deposition and promoted cell apoptosis.	MiR-7-5p/Epac1	[91]

*CF* Cardiac fibrosis, *Nlgn* Neuroligin, *Slc8a1* Solute carrier family 8 member A1, *IL* Interleukin, *EGR* Early growth response, *TAC* Transverse aortic constriction, *YAP1* Yes-associated protein 1, *TPM4* Tropomyosin-4, *ACTG* Gamma-actin, *ATBP* ATP synthase 19 subunit B, *COX5B* Cytochrome c oxidase, *NUDFS2* NADH dehydrogenase iron sulfur protein 2, *ATP* Adenosine triphosphate, *Ube3a* Ubiquitin protein ligase E3A, *M2M* *M2* Macrophage, *SEV* Small extracellular vesicles, *RhoC* Ras homolog gene family,member C, *RSF1* Remodeling and spacing factor 1, *RAC1* Ras-related C3 botulinum toxin substrate 1, *HF* Hepatic fibrosis, *FBXW4* F box and WD 40 domain containing protein 4, *PSD3* Pleckstrin and Sect. 7 domain-containing 3, *HSC* Hepatic satellite cell, *NASH* nonalcoholic steatohepatitis, *SCAR* Steatohepatitis-associated circRNA ATP5B regulator, *ROS* Reactive oxygen species, *ATP5B*, *ATP* synthase subunit beta, *mPTP* Mitochondrial permeability transition pore, *CypD* Cyclophilin D, *IPF* Idiopathic pulmonary fibrosis, *ANKRD42* Ankyrin repeat domain 42, *ECM* Extracellular matrix, *AJUBA* Lim domain-containing protein ajuba, *TADA2A* Transcriptional adaptor 2 A, *Cav* Caveolin, *HIPK3* Homeodomain-interacting protein kinase 3, *COL1A1* Collagen1A1, *TGFb1* Transforming growth factor-β, *SOX4* SRY-related high-mobility-group box 4, *FOXK* Forkhead box K, *FAN* Folic acid induced nephropathy, *FN* Fibronectin, *RF* Renal fibrosis, *UUO* Unilateral urethral obstruction, *IRI* Ischemia reperfusion injury, *PF* Pulmonary fibrosis, *BNC2* Basonuclin 2, *TEC* Tubular epithelial cell, *CDK* Cyclin-dependent kinase, *FUSE* Far upstream element, *FUSP1* Far upstream element binding protein 1, *GAB1*, *GRB2* associated binding protein 1, *Epac1* Exchange protein directly activated by cAMP 1

#### Cardiac fibrosis

Cardiovascular diseases remain the leading cause of death worldwide, causing nearly 18 million deaths annually [42]. Cardiac fibrosis represents a unifying theme across various etiologies, including coronary artery disease, myocardial infarction, and diabetes mellitus. Myocardial fibrosis can be divided into reactive or replacement fibrosis based on the pathological manifestations or interstitial, perivascular and subendocardial fibrosis, according to the locations of ECM deposition [43]. Accumulating evidence has shown that circRNAs are involved in cardiac fibrosis [44]. Doxorubicin, an extensively used chemotherapeutic drug, induces cardiac remodeling manifesting as cardiofibrosis, hypertrophy, dilation, cardiomyopathy, and decreased contractile strength due to its pronounced cardiotoxicity. circNlgn is highly abundant in the heart, whereas its parental gene Nlgn is hardly detectable. The translated product of circNIgn–Nlgn173 mediates the side effect of doxorubicin-induced cardiac fibrosis by binding to and activating H2AX, subsequently upregulating inflammatory cytokines, including IL-1b, IL-2Rb, and IL-6, and early growth response molecules [45]. The increased nuclear localization of circNIgn is mediated by interacting with the structural protein LaminB1 through its unique 9-amino-acid motif, which ultimately leads to aberrant collagen deposition, cardiac fibroblast proliferation, and reduced cardiomyocyte viability [46]. Another highly conserved circRNA insulin receptor (circ-INSR) protects against doxorubicin-mediated cardiotoxicity and represses cardiac remodeling by cooperating with single-stranded DNA-binding protein 1, which improves mitochondrial function by stabilizing mtDNA [47]. A 208-amino acid protein derived from circ_0036176 (back-splicing from exon 2 to exon 4 of myosin IXA) inhibits the proliferation of cardiac fibroblasts by suppressing the cyclin/retinoblastoma tumor-suppressor gene pathway, whereas miR-218-5p can bind to circ_0036176 and inhibit its translation, thus abolishing the effect of circ_0036176 on inactivating cardiac fibroblasts [48]. Together, circNlgn, circ-INSR and circ_0036176 are representative circRNAs that can be translated into novel isoforms and confer unique functions beyond their parental genes, suggesting that these coding circRNAs and derived peptides are potential therapeutic targets for cardiac injury and fibrosis.

CircRNAs also modulate cardiac fibrogenesis by interacting with key proteins, affecting m6A modification, or via exosomes. circYap is downregulated in cardiac diseases, and ectopic expression of circYap significantly improves cardiac function and mitigates cardiac fibrosis. Mechanistically, circYap binds to gamma-actin and tropomyosin-4 (TPM4) and increases the inhibitory effect of TPM4 on actin polymerization, thus exerting an antifibrotic role in cardiac tissue [49]. circZNF609 regulates the expression of yes-associated protein (YAP) by balancing the recognition of the m6A reader protein YTHDF by Yap mRNA, thus affecting Hippo-YAP and Akt signaling. Downregulation of circZNF609 exhibits a cardioprotective effect and combats myocardial remodeling [50]. Extracellular circRNAs can also serve as messengers mediating crosstalk between different cell types in myocardial remodeling [51]. CircUbe3a from M2 macrophage-derived small extracellular vesicles promotes the activation, migration, and phenotypic transformation of cardiac fibroblasts via the miR-138-5p/RhoC signaling pathway, which ultimately aggravates cardiac fibrosis after acute myocardial infarction [52]. These studies highlight the diverse functions of circRNAs in cardiac remodeling and provide potential targets for antifibrosis therapy.

Recently, Yang et al. attempted to explore the effect of circRNA-based therapy in a mouse model by using circularized antisense RNA to block sense circslc8a1. circslc8a1 is the most abundant circular RNA in the mouse heart and is significantly decreased 8 to 12 weeks after transverse aortic constriction surgery. Silencing cardiac-specific circslc8a1 by its antisense induces cardiac hypertrophy, fibrosis and hepatic steatosis, and overexpressing circslc8a1 in vivo improves heart function and alleviates hepatic steatosis [53]. This study provided the foundation for potential clinical applications in circular RNA therapy.

#### Liver fibrosis

Chronic liver diseases, including viral infections (such as hepatitis B and C), autoimmune hepatitis, alcoholic steatohepatitis, nonalcoholic steatohepatitis, and progressive metabolic diseases, gradually result in liver dysfunction and fibrosis. Eventually, fibrosis progresses into advanced stage cirrhosis. Patients with decompensated cirrhosis develop life-threatening complications, and most die within a median time of approximately two years [54]. Despite increasing mortality and morbidity due to hepatic fibrosis, there are no approved antifibrotic therapies, and liver transplantation is currently the only treatment option [55]. Therefore, there is an exigent need to clarify the cellular and molecular mechanisms underlying hepatic fibrosis. Recent studies have revealed functional roles for circRNAs in liver fibrosis [56], most of which are focused on their role as miRNA sponges in hepatic stellate cells (HSCs). For example, circular RNA RSF1 promotes HSC proliferation and proinflammatory and profibrotic phenotype changes by targeting the miR-146a-5p/RAC1 axis [57]. Hsa_circ_0070963 [58] and circFBXW4 [59] inhibit liver fibrosis by sponging miR-223-3p and miR-18b-3p, thus upregulating LEMD3 and FBXW7, respectively. CircRNA derived from the Pleckstrin and Sect. 7 domain-containing 3 (PSD3) gene (circPSD3) is significantly downregulated in HSCs and liver tissues of mice in the CCl4-induced mouse model of liver fibrosis. Loss-of-function and gain-of-function analyses suggest that circPSD3 inhibits the activation and proliferation of HSCs by acting as a sponge for miR-92b-3p and subsequently promotes the expression of Smad7 [60]. Another circRNA derived from the MTO1 gene, namely, cMTO1, sponges miR-181-5p and enhances the expression of PTEN in HSCs, thereby exerting an antifibrotic role [61]. Additionally, hsa_circ_0004018 [62], circ608 [63], circCREBBP [64], and circUbe2k [65] suppress or promote hepatic fibrosis in the same manner as miRNA sponges.

Exosomal circRNAs could serve as messengers, disease biomarkers or therapeutic targets in liver fibrosis. circCDK13 packaged in human bone marrow mesenchymal stem cell-derived exosomes inhibits the activation and proliferation of HSCs and attenuates liver fibrosis in vivo. Mechanistically, exo-circCKD13 promotes the transcription of MFGE8 by targeting miR-17-5p/lysine acetyltransferase 2B and inhibits the PI3K/AKT and NF-κB signaling pathways [66]. mmu_circ_0000623 expression is downregulated in CCl4-induced fibrotic mouse livers, and mmu_circ_0000623-modified adipose-derived mesenchymal stem cell exosomes suppress CCl4-induced liver fibrosis by promoting autophagy through the miR-125/ATG4D axis [67]. Transfer of mesenchymal stem cell-derived exosomal circDIDO1 suppresses HSC activation through the miR-141-3p/PTEN/AKT pathway, providing a potential therapeutic target [68].

Groundbreaking research by Zhao et al. showed that although circRNAs transcribed by the mitochondrial genome account for less than 0.1% of overall circRNomics, they contribute nearly 40% of the downregulated circRNAs in fibroblasts from livers with nonalcoholic steatohepatitis [69]. Mitochondrial-localized steatohepatitis-associated circRNA ATP5B Regulator (SCAR), which is regulated by PGC-1α, binds to ATP5B. SCAR suppresses mitochondrial permeability transition pore opening and inhibits the release of reactive oxygen species and collagen contraction in HSCs. Interfering with this mito-circRNA mitigates high-fat diet-induced cirrhosis and insulin resistance in mice, thus serving as a therapeutic target.

#### Pulmonary fibrosis

Pulmonary fibrosis is an age-related, progressive and fatal disease with a median survival time of 3–5 years after diagnosis [70]. Chronic obstructive pulmonary disease, idiopathic pulmonary fibrosis, asthma, cystic fibrosis, and silicosis are common etiologies of pulmonary fibrosis. Various types of circRNAs have been discovered to be differentially expressed and involved in the pathogenesis of lung fibrosis. circANKRD42 promotes the nuclear translocation and translation of YAP1 through the miR-324-5p/AJUBA and miR-136-5p axes, mediating the crosstalk between mechanical stiffness and biochemical signals in lung fibrosis [71]. CircRNA TADA2A retards pulmonary fibrosis by targeting miR-526b and miR-203 and upregulates Caveolin (Cav)-1 and Cav-2, thereby inhibiting fibroblast proliferation and activation [72]. Wang and colleagues reported increased m6A-circRNA modifications in a silicosis mouse model. They identified highly m6A-modified hsa_circ_0000672 and hsa_circ_0005654 as the major targets of methyltransferase-like 3. A series of functional experiments in vitro further confirmed the fibrogenic nature of these two circRNAs [73]. circHipk3 is upregulated in TGF-β1-stimulated fibroblasts, and silencing circHipk3 abrogates the activation, proliferation, and glycolysis of fibroblasts in vitro. By targeting miR-30a-3p and promoting the expression of forkhead box K2, circHipk3 facilitates fibroblast glycolysis and activation in lung fibrosis [74]. Zhang et al. also demonstrated that by sponging miR-338-3p, circHipk3 increases the expression of SRY-related high-mobility-group box 4 and collagen 1A1, therefore promoting the fibroblast-to-myofibroblast transition [75]. Although the existing studies on circRNAs in lung fibrosis have mainly focused on their typical miRNA sponge role, they tell the story from novel perspectives, including mechanical stiffness, m6A modification and metabolic aspects.

#### Renal fibrosis

CKD affects more than 10% of the world’s population, and renal fibrosis is the common endpoint of almost all progressive kidney diseases of various etiologies. Renal fibrosis is a complicated, irreversible and detrimental process that affects not only the kidney but also other organ systems, including the cardiovascular system, bone and digestive systems. Renal fibrosis is initiated at certain focal sites following injurious stimuli, gradually forming a specialized microenvironment that triggers fibroblast activation and ECM deposition [76]. ECM–cell interactions integrate various fibrogenic signal inputs and trigger intracellular signal propagation to produce ECM components as well as their extracellular assembly, forming a vicious cycle. Thus, a better understanding of the complex cellular and molecular events could help us to improve antifibrotic therapies for renal fibrosis. Accumulating evidence has implicated circRNAs in the pathogenesis of renal fibrosis [77, 78]. For instance, circ_0000064 is upregulated in mesangial cells from high glucose (HG)-induced renal fibrosis and in kidneys from patients with diabetic nephropathy. circ_0000064 promoted HG-induced proliferation, inflammation and ECM accumulation of mesangial cells in vitro by targeting miR-424-5p and promoting the expression of WNT2B [79]. circ_0000064 expression is also increased in the serum of patients with diabetic nephropathy and HG-induced HK-2 cells. Silencing circ_0000064 attenuates HG-induced HK-2 cell injury by targeting the miR-532-3p/ROCK1 axis [80]. Overexpression of circHIPK3 aggravates folic acid-induced renal tubulointerstitial fibrosis by sponging miR-30a, subsequently activating TGF-β1 signaling in HK-2 cells [81]. However, circHipk3 has a distinct expression pattern and mechanism of action in different kidney diseases and cell types. According to Zhuang et al., circHipk3 is downregulated in HG-induced HK-2 cells and ameliorates the inhibition of proliferation and apoptosis induced by HG. By targeting miR-326 and miR-487a-3p, circHipk3 increased the expression of SIRT1, therefore alleviating HG toxicity [82]. The expression of circHIPK3 in kidney tissues of patients with diabetic nephropathy and mesangial cells in HG stimulation is increased significantly, and knockdown of circHipk3 inhibits cell proliferation and increases the mRNA levels of profibrotic markers, including TGF-β1, Collagen 1 and fibronectin, by sponging miR-185 [83]. Epithelial cell cycle arrest at G2/M is one of the major driving forces in the development and progression of renal fibrosis. Wang et al. identified a kidney- and liver-enriched DEAH-box helicase 9-induced circRNA, circBNC2, which promotes the formation and nuclear translocation of CDK1/cyclin B1 complexes and combats G2/M arrest. Restoring the downregulated circBNC2 in kidneys with ischemic reperfusion injury mitigates ECM deposition and renal fibrosis [84]. circPTPN14 was identified as a scaffold enhancing the binding between far upstream element (FUSE) binding protein 1 and FUSE, and the tertiary structure reinforced the transcription of MYC and related fibrotic genes, which exacerbated renal fibrosis [85].

Intriguingly, circRNAs have been identified in exosomes secreted by the kidneys and are ideal candidates as biomarkers and therapeutic targets in kidney diseases. Cao et al. detected exosomal circRNA profiles using urine samples from patients with CKD with or without renal fibrosis. According to microarray data, hsa_circ_0036649 may be a fibrosis-related circRNA. The authors further verified urinary exosomal hsa_circ_0036649 expression in 110 patients with biopsy-proven CKD and 54 healthy controls. hsa_circ_0036649 expression was significantly decreased in patients with renal fibrosis and correlated with kidney function and the degree of fibrosis [86]. Another urinary exosomal circRNA, hsa_circ_0008925, was elevated in patients with renal fibrosis, and its level was associated with serum creatinine, blood urea nitrogen, estimated glomerular filtration rate, and cystatin C, as well as pathological scores for tubulointerstitial fibrosis and glomerular sclerosis [87]. Exosomal circ_DLGAP4 was increased in HG-treated mesangial cells from rat models and from patients with diabetic kidney disease and significantly promoted the proliferation and fibrosis of mesangial cells. Mechanistically, circ-DLGAP4 acted as a sponge of miR-143 and activated the Erb-b2 receptor tyrosine kinase 3/NF-κB/MMP-2 axis [88]. Although most of these studies are relevant and descriptive, they pave the way for the applications of circRNAs as biomarkers in easily accessible clinical biospecimens, such as blood or urine samples from patients with kidney diseases.

#### Skin fibrosis

Keloids are elevated, itchy, painful, and cosmetically disturbing fibroproliferative scars that arise from excessive wound healing that involves healthy surrounding skin. Keloids may significantly hamper patients’ psychological well-being and daily functioning, resulting in a heavy burden that greatly affects their quality of life. However, the specific pathogenesis of keloids remains to be fully elucidated. circRNAs contribute to the etiology of keloids by affecting keloid fibroblasts. Circ_0057452 sponges miR-7-5p and upregulates GRB2-associated binding protein 1, thereby affecting the proliferation, apoptosis, migration, invasion, collagen synthesis and cell cycle arrest of keloid fibroblasts [89]. Circular nuclear receptor interacting protein 1 (circNRIP1) is upregulated in keloid tissue and keloid fibroblasts. Loss-of-function analysis showed that circNRIP1 facilitated the proliferation and ECM deposition and inhibited the apoptosis of keloid fibroblasts. By repressing Fox4-mediated Fragile-X mental retardation 1 (FXR1) ubiquitination and degradation, circNRIP1 induces the maturation of premiR-503 and increases miR-503-3p and miR-503-5p. The circNRIP1/FXR1 miR-503-3p and miR-503-5p axes promote keloid fibroblast proliferation and fibrosis [90]. circCOL5A1 promotes the growth and ECM accumulation of keloids in vitro and in vivo by targeting exchange proteins directly activated by cAMP, acting as a sponge of miR-7-5p and affecting the PI3K/Akt signaling pathway [91].

### CircRNAs as antifibrotic therapeutic targets and fibrotic biomarkers

The aberrant expression and functional roles of circRNAs suggest that they are not simply splicing byproducts but are modulators of numerous physio-pathological processes. Interfering with circRNAs may provide a new strategy for RNA therapy, and the advantages and disadvantages of different methods of interference are provided in Table 2. Common strategies to overexpress circRNAs include using plasmid or synthetic circRNAs, and small interfering RNAs/small hairpin RNAs, antisense. Cre/lox system, or CRISPR/Cas systems are utilized to downregulate circRNAs. Furthermore, circRNAs can be packaged into engineered nanoparticles, adenovirus vectors, or exosomes to be delivered in animal models in vivo. Synthetic circRNAs have been explored for their application as a novel class of vaccines [92]. circRNA-based anticancer therapy ideally achieves high levels of stability, high protein expression rate and robust immune activation, thus showing the most potential for application in a wide range of hard-to-treat malignancies [93]. There are still some challenges in developing circRNA-targeted therapies, including the off-target effects of circRNA interference, immunogenicity triggered by synthetic circRNAs, safety of the encapsulated nanoparticles, and nonspecific expression patterns. With the help of single-cell sequencing technology, more disease-, tissue-, and cell-specific circRNAs will be identified at a high resolution, and new interfering approaches, including using antisense circRNAs, CRISPR/Cas systems and safer packaging technologies, will provide robust evidence for future circRNA-targeted therapies [94].

**Table 2:** The advantages and disadvantages of different methods targeting circRNA therapeutics

Pattern	Technology	Material	Effect	Advantages	Disadvantages
In vitro	RNAi	ShRNA, siRNA, ASO.	KD	1. Convenient to synthesis and deliver; 2. Relative low expense.	1. Rapid degradation; 2. Low delivery efficiency; 3. Lack of cell-specificity; 4. Off-target effect.
In vivo &vitro	CircRNA expression vector	Plasmid, LV, AAV.	OE & KD	1. Relative stable; 2. Frequently used.	1. Mis-spliced product; 2. Off-target effects.
In vivo & vitro	Synthetic circRNA	Circularized RNAs	OE	Highly purified.	1. Difficult to generate large amount; 2. Immune system activation.
In vivo	Nanoparticle	Liposome, polymer, dendrimer, inorganic material (gold, or metal oxides).	OE & KD	1. Target specific cell through receptors and ligands; 2. Relatively stable from degeneration; 3. Facilitate cell uptake; 4. Prevent to immune activation.	1. Limited to target to the cytoplasmic circRNAs; 2. Low delivery efficiency; 3. Toxicity.
In vivo &vitro	Exosome	Exosome	OE & KD	1. Protect RNAi molecules from degradation; 2. Promote cellular uptake; 3. Biocompatible.	1. Difficult in manufacturing; 2. High cost.
In vivo	Cre-lox system	Cre recombinase	KO	1. Cell-type specific or tissue specific; 2. Relatively safe.	1. Ectopic Cre expression leads to the off-target effect; 2. Need validation of genotyping.

*RNAi* RNA interfering, *shRNA* Short hairpin RNA, *siRNA* Short interfering RNA, *ASO* Antisense oligonucleotides, *LV* Lentivirus, *AAV* Adenovirus-associated virus, *OE* Overexpression, *KD* Knock-down, *KO* Knock-out

Resistance to exonuclease degradation endows circRNAs with ideal biostability and high pharmaceutical stability. An increasing number of circRNAs have been detected in body fluids, such as plasma, serum, urine, and cerebrospinal fluid [95]. circRNAs are utilized as biomarkers to discriminate cancer patients from healthy individuals [96]. Their translational value as biomarkers in fibrosis deserves clarification in future studies.

## Conclusion

Fibrotic remodeling impairs organ function and represents a real challenge for human public health. The past decade has witnessed fruitful progress in the understanding of the functions of circRNAs in fibrotic diseases. They play pivotal roles in fibrotic tissues and organs through distinct modes of action, far beyond the early-discovered miRNA sponge role. However, challenges and difficulties posed by theoretical deficiencies and technological restrictions exist. Future studies of circRNAs should extend the entire research direction from association and description studies to mechanistic exploration and focus on other important cell types, such as epithelial, endothelial and immune cells.

Understanding the steps by which dysregulated circRNAs drive fibrosis development can aid in the development of specific targets of its cascading mechanism. For example, although fibrosis is a multistaged and highly dynamic process triggered by various risk factors and inflammatory responses, there are common core and regulatory fibrotic pathways shared by different organ systems. Previously, directly targeting TGF-β failed, as this vital molecule helps to regulate homeostasis under physiological conditions. However, identifying circRNAs that activate or repress a highly consistent set of ligands and receptors and fibrotic pathways that promote tissue and cell injury, inflammatory responses, myofibroblast activation and ECM remodeling will pave the way toward the delivery of specific and effective antifibrotic therapies in the future. Meanwhile, circRNAs are promising biomarkers for the diagnosis and prognosis of organ fibrosis due to their specificity, abundance, and biostability.

## Data Availability

Not applicable.

## Abbreviations

circRNAs: Circular RNAs
ECM: Extracellular matrix
miRNAs: MicroRNAs
RBPs: RNA binding proteins
ciRNAs: Intronic circrnas
EIciRNAs: Exon‒intron circRNAs
mecciRNAs: mitochondrial circRNAs
snRNP: Small nuclear ribonucleoprotein
ROS: Reactive oxygen species
Ago2: Argonaute 2
RISC: RNA-induced silencing complex
TGF-β: Transforming growth factor-β
smad: Solvated metal atom dispersed
PI3K: Phosphatidylinositol 3-kinase
PDGF: Platelet-derived growth factor
TPM4: Tropomyosin-4
YAP: Yes-associated protein
HSCs: Hepatic stellate cells
FUSE: Far upstream element
FXR1: Fragile-X mental retardation 1
